# Engineering of injectable hydrogels associate with Adipose-Derived stem cells delivery for anti-cardiac hypertrophy agents

**DOI:** 10.1080/10717544.2021.1943060

**Published:** 2021-06-28

**Authors:** Guangyu Long, Quanhe Wang, Shaolin Li, Junzhong Tao, Boyan Li, Xiangxiang Zhang, Xi Zhao

**Affiliations:** a Department of Cardiology, Zhengzhou Central Hospital Affiliated to Zhengzhou University, Zhengzhou, China; b Department of Cardiology, The First Affiliated Hospital of Zhengzhou University, Zhengzhou, China

**Keywords:** Adipose-derived stem cells, Injectable hydrogels, alginate sericin laminin, anti-cardiac, hypertrophy

## Abstract

Adipose-derived stem cells (ADSCs) treatment offers support to new methods of transporting baseline cell protein endothelial cells in alginate (A)/silk sericin (SS) lamellar-coated antioxidant system (ASS@L) to promote acute myocardial infarction. In the synthesized frames of ASS, the ratio of fixity modules, pores, the absorption and inflammation was detected at ka (65ka), 151 ± 40.12 μm, 92.8%, 43.2 ± 2.58 and 30.10 ± 2.1. In this context, ADSC-ASS@L was developed and the corresponding material was stable and physically chemical for the development of cardiac regenerative applications. ADSC-ASS@L injectable hydrogels in vitro examination demonstrated higher cell survival rates and pro-angiogenic and pro-Inflammatory expression factors, demonstrating the favorable effect of fractional ejections, fibre-areas, and low infracture vessel densities. In successful cardiac damage therapy in acute myocardial infarction the innovative ADSC injection hydrogel approach may be helpful. The approach could also be effective during coronary artery hypertrophy for successful heart damage treatment.

## 1. Introduction

Approximately 10 million cardiovascular deaths globally are due by acute myocardial infarction (AMI). Coronary physiologies supply nutrients and oxygen to the whole vessels of the body. The AMI is due to necrosis of the cardiomyocytes due to myocardial ischemia because of the variance in blood flow and myocardial injury (Wang et al., 2009; Li et al., 2018; Li et al., 2020; Vignoli et al., 2020). The principal goal of AMI therapeutic attention is hence to improve blood and myocardial recovery regulation. Cardiovascular treatments include enzyme inhibitor vasodilators, anticoagulants and antiplatelet characteristics. All drugs for patients are carefully treated but may be associated with numerous serious side effects (Liu et al., 2016; Su et al., 2018; Kaya et al., 2020; Fu et al., 2020; Li et al., 2020).

In tissue nanotechnology, hydrogels are significant. They show a local tissue ecology which strives to create new organic and biochemical functionalities. They include the development of nanostructures in polymers, ceramics, nanotubes and graphs (Fan et al., 2017; Cui et al., 2018; Wang et al., 2019; Liang et al., 2019; Kim et al., 2020). Hydrogels are therefore employed to intensely connect ligand re-organized cellular matrix ligation proteins in diverse cell types with the cell membrane protéins for cell encapsulation, transplantation, and diverse tissue application processes. Collagen fibrils may have a position in the cell adhesion of alginates and tissue-engineered tissue hydrogels (Melhem et al., 2017; Yuan et al., 2019; Lyu et al., 2020). Often alginate hydrogels are missing from the fibrous matrix of local tissue (Wang et al., 2017). An indigenous connection to the RGD site target in the cell adherence process. Finally, a conventional nanotopographic piece of collagen that differs from collagen (Tous et al., 2011; Paul et al., 2014; Tang et al., 2017; Lin et al., 2020).

The adipogenesis-derived bone marrow, embryo, myocardial mesenchymal and hemotoprosthetic stem cells (ADSCs) have developed from a range of points of view. Though cells' varied views enhance the heart, the heart tissue is not exact (Han et al., 2019; Gao et al., 2020; Arzaghi et al., 2021). Consequently, numerous stem cell research have reduced cardiac stem cell heart tissue distortion because similar difficulties arise up to and have no results. Soft cell configuration is critical for the innate processes of immunity and immunological suppression (Li et al., 2012; Chen et al., 2020; Feng et al., 2020). We have produced ADSCs for laminated alginate sericin in this investigation to enhance heart tissue. This injectable ADSC-ASS@L scaffolding device permits the angiogenesis gene to protect the heart tissue against heart failure.

## 2. Methods and materials

### 2.1. Extraction of sericin

Sericin was collected for degumming at high temperatures utilizing the above-mentioned procedures. The cocoon was filtered off and cleaned with water. During 1 hour and 10 ml DD-water, the little bits were divided and dissolved at 100 °C. Fibroin was filtered into 15 kDa baASS with the focus on 10000 PEGs, then put on a rotary evaporator and dialyzed for 1 day. At −20 °C, the final compounds were removed and congealed, and lyophilized white solids.

### 2.2. Preparation of alginate -sericin (ASS) hydrogels

ASS frame was developed using the hydrogeling technology. A 2:1 alginate combination and sericin were utilized in water solution. In addition, the primary portion of jelly and sericin was dipped in DD water at 70 °C and slowly cooled. 0.8% of the cross-linking system was then used in the same technique and the products unreacted were cleansed. Finally, we used dried companies for more applications. Moral testing was conducted thereafter to do the rigorous SEM and TEM analysis.

### 2.3. Characterization techniques

The hydrogels were prepared, and one drop of hydrogels was mounted on the carbon-coated TEM grids at room temperature (RT) during HR-TEM. The size and distribution ratio were measured using 55 readings from the TEM photographs (TECNAI-TEM, USA). The structural properties were analyzed using HG, and the ADSC-HASS were examined via FT-IR.

### 2.4. Isolation of ADSCs

The previously described method (Subarkhan & Ramesh, 2016; Mohamed Subarkhan et al., 2016; Mohan et al., 2018) has been used to achieve the ADSCs. First, Wister Mice weighed around 19–22 g and 6-week old female. The rats were intraperitoneal and incised and ketamine (80 mg/kg) were anesthetized. Collagenase was used to collect and consume the mammalian fat bowl for one hour at 37 °C. The digest has been cured using a netting of 100 μm. Five minutes at 1500 rpm, and 10% FBS DMEM was removed with 1% antibiotic from the digester. The digester was centrifuged. An estrology medium was also employed to assess the probable variance of isolated cells when isolating the isolated cells from the estrogen's.

### 2.5. Animal model and experimental groups for myocardial infarction

Myocardial infarction was started using the previously mentioned technique (Aden et al., 2015; Noshadi et al., 2017; Liu et al., 2018; Budharaju et al., 2021). The institute's animal welfare unit's methodologies were applied in the animal treatment and experimental methodology. Fisher rats weighing 200–250 g were obtained from the Chinese Medical University (eight animals per group). In a sterile region, there were four universities and animals per enclosure. AMI was exposed to all treatment classes, as outlined in the literature. These tests employed selectively injected rats to differentiate the animal classes. During the experiment, five groups were employed, and data is supplied. Group I was not given any treatment (healthy animals). With a 30 mm syringe needle, Group II was injected with saline and unlimited ADSC-ASS (0.5 g/L). Group III was injected with ADSC-ASS@L (0.5 g/L). ADSCs (5 105 cells per 100 L saline infused into the ADSCs) were injected into Group IV. The samples were successfully generated and recognized via the bleb on the infarction site utilizing an infringed diet. Every three days, the rat's climate-controlled and sterile rooms were inspected using simple asses to feed.

The Fisher rats were sacrificed at the end of the testing. The heart and left ventricle wet weights were calculated and normalized to the animal's body weight. The left ventricular tissue samples were soaked in 4% paraformaldehyde, embedded in paraffin, and serially sectioned (5–6 m sections). For the cross - section areas of cardiac myocyte and collagen deposition, the slides were stained with hematoxylin and eosin (HE) or Masson trichrome (MTS), respectively. The remaining pieces were flash-frozen in liquid nitrogen for real-time reverse transcription polymerase chain reaction (RT-PCR) analysis.

### 2.6. Statistical analysis

Statistical differences were achieved using oneway analysis of variance (ANOVA) followed by Tukey’s test with GraphPad Prism 8 software (GraphPad Software Inc., La Jolla, CA). For all tests, *p-value <.05, **p-value <.01, and ***p-value <.001.

## 3. Results and discussion

### 3.1. Fabrications and description methods of ADSC-ASS@L hydrogels

**Figure 1.**
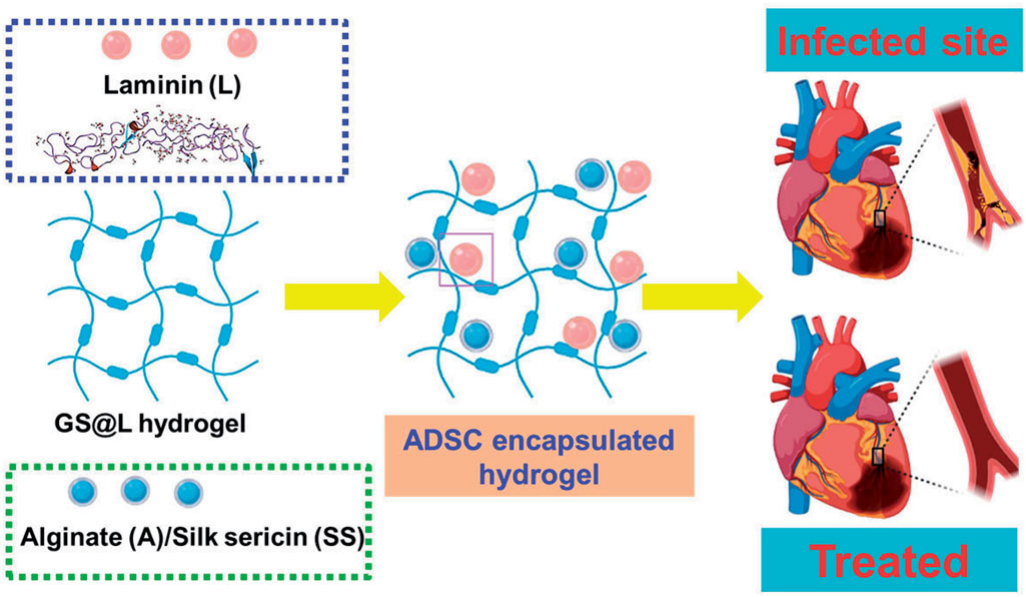
(A) schematic representation of hydrogels fabrications for myocardial infractions.

Hydroglation and hydrogels containing sericin substituents were made using hydroglation procedures (Garbayo et al., 2021; Zhao et al., 2021; Shi et al., 2021; Yu et al., 2021). The solidification of an aqueous solution at 0 °C suggests polymerization, and the results were in polymer and water isolation. The hydrogels were massive, especially at high polymer concentrations with the crosslinker (Figure 1).

Having frozen the hydrogels, removed solids and got the pores that were produced by the polymers. The SEM figure shows that hydrogels exhibit precise forms with basic porosity properties, such as sheet morphologies. Additionally, we studied the morphology of the hydrogels, which exhibited extremely porous hydrogels (Mukherjee et al., 2010; Navaei et al., 2017; Liu et al., 2018). Hydrogels enhance porosity from 85 to 90% with the inclusion of sericin into ASS versions. The actual porosity size of the moieties of Alginate was 150–200 microns. This porous scale is suitable for the integration of fibroblasts and stem cells. In dry as well as in wet conditions, the hydrogel density model was established, and sericin was applied to alginate to decrease the density model. This may be due to a 5% increase in pores. Under moist conditions, the Alginate model had 72 kPa, but the ASS model had 65 kPa. These parameters are slightly like the overall importance of the previously mentioned tissue regeneration model.

### 3.2. Swelling and dehydration properties of hydrogels

**Figure 2.**
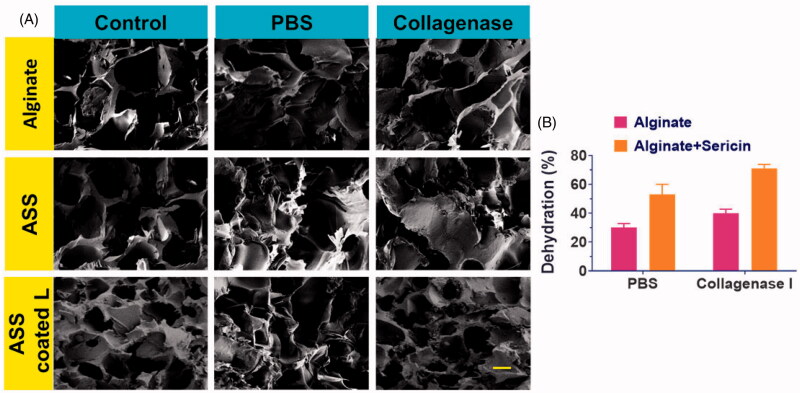
(A) The SEM analysis of synthesized hydrogels displaying pores and size of the porous, the scale bar is 200 µm. The SEM analysis with various composition are displayed (with PBS and collagenase). (B) The bar diagram represents the quantitative data of the Alginate and Alginate-Sericin frameworks dehydration associated to the PBS and collagenase at day 7.

In order to design molds, the swelling of hydrogels is crucial. The porous cross linker troughs the same vehicles, gases, and nutrients for the survival of a specific cell. The sales have been examined by hydropowered suppliers. The expanding holes could boost the system's prolonged penetrability after combination of sericin (Wang et al., 2009; Song et al., 2016; Zhang et al., 2019). The frameworks were evaluated in the event that the collagenase enzyme was in conformity with the above although they may be used for skin strengthening. This comprises 70% alginate sericin and 45% frameworks of alginate, but the enzyme deprivation of PBS is small and about 39% alginate sericin and 25% alginate frameworks on the seventh day have been disposed of, as is the case for mass losses after growing, due to massive loss in frameworks (Figure 2(B)). The absorbing pore mounting fall and breakdown in any category can be seen in SEM pictures (Figure 2(A)). But the agreement was in all communities. However, under the two situations in the ASS system, structural decay was obvious (Figure 2(B)). The whole poverty of frames was noticed throughout day 14, thanks to the dual Alginate e- and ASS frameworks. The off-site well of the myocardial infarction mechanism should be cleared because of these benefits in hydrogel frame examination.

**Figure 3.**
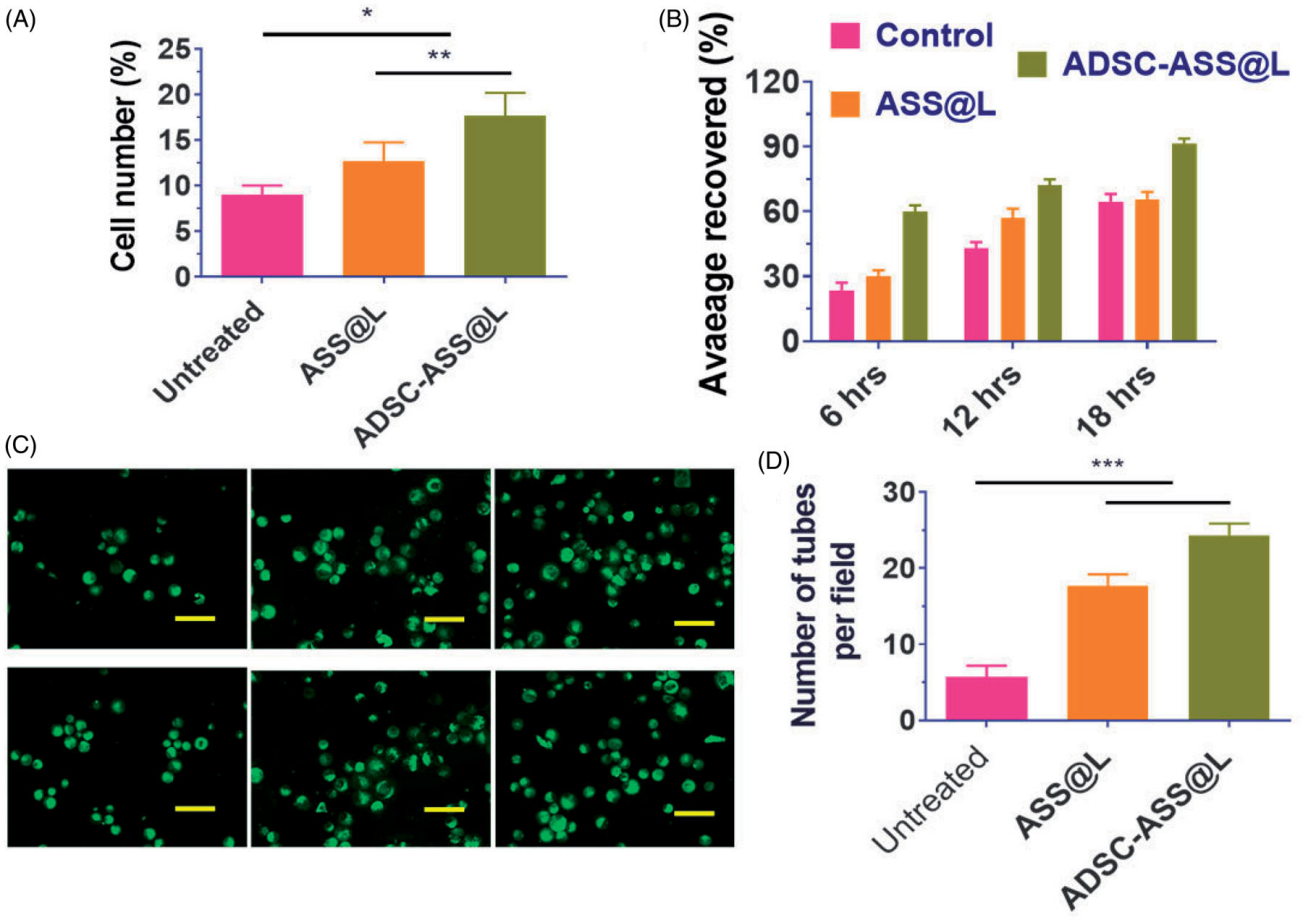
The bio-compatibility and potentials of angiogenic ADSCs with fabricated injectable hydrogels in vitro framework. (A) In vitro quantitative analysis cell proliferations by the MTT assay of the ADSCs. (B) Quantitative examination of area average covered (cell migration) by ADSCs in different time stages (6, 12 and 18 hrs. (C) Flurocence microscopy investigation of cell proliferations and tube formations with prepared hydrogels medium after 24 h of incubations. Scale bar 100 µm. (D) The quantitative determinations of tube formation after 24 hrs. *p-value <.05, **p-value < .01, and ***p-value < .001.

Bioavailability and adequacy of the ADSC-ASS@L hydrogels produced have been established in vitro studies. In the research of proliferation and viability of cells, the usage of human coronary endothelial cells (HCAECs) grown on synthetic hydrologists ADSC-ASS@L was utilized and their ratios estimated in Figure 3(A–D). Clearly, the cellular proliferation of HCAEC cells in hydrogels from aDSC-ASS@L was substantial. The results indicate that, in comparison to the free ASS@L, ADSC-ASS@L hydrogels have a significant percentage of survival and proliferation on cells. The effect of the hydrogels at 6 h and 12 h was not observed by cell proliferation. However, the proliferation percentage significantly increased in the treated groups after 18 h.

**Figure 4.**
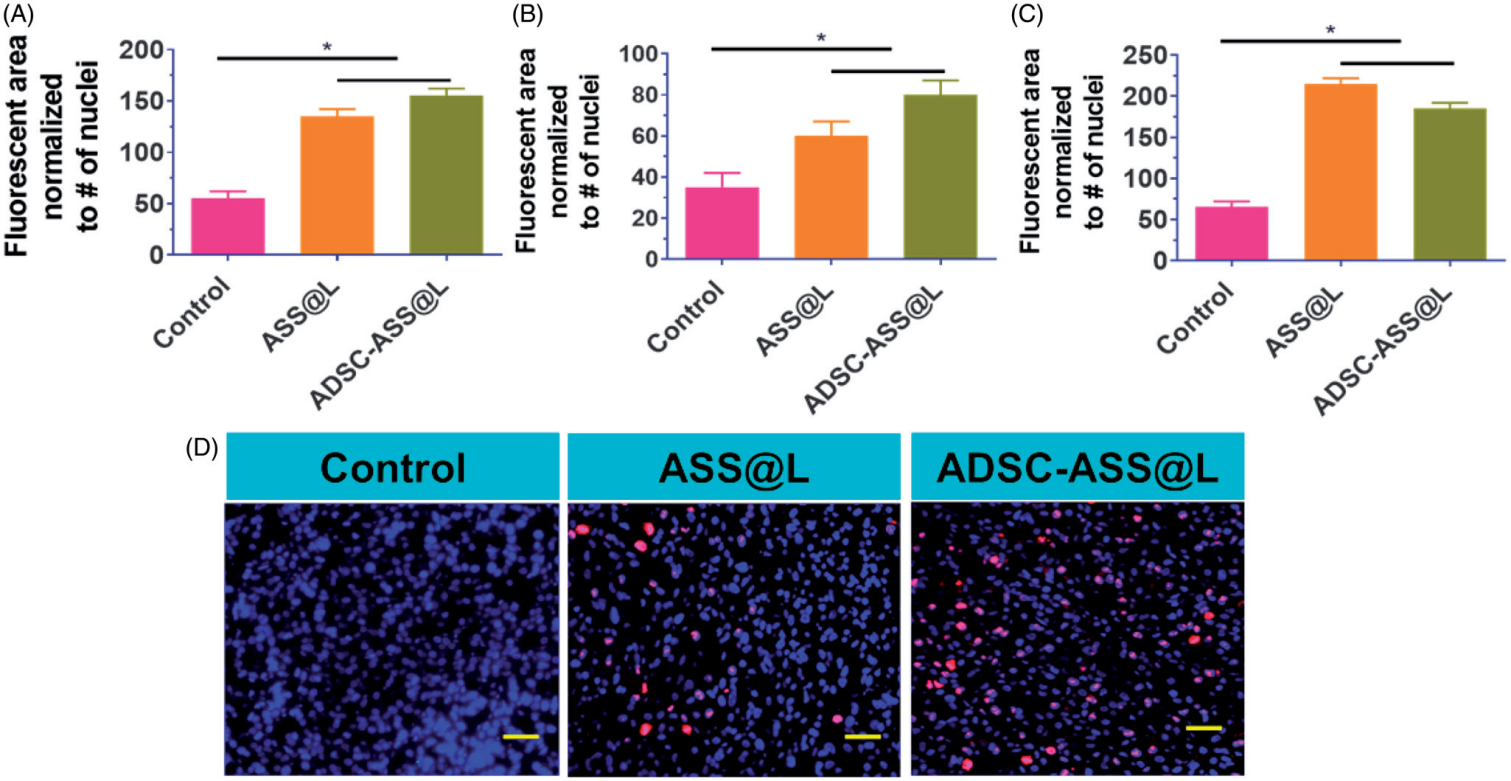
(A–C) The cardio markers expression Cx43 early stage in vitro treatment with ASS@L and ADSC-ASS@L. D) The Cx43 fluorescence microscopy investigation of ASS@L and ADSC-ASS@L. Scale bar 20 µm. *p-value <.05.

We also examined the cardiomyocytic proliferation factors of the ADSC-ASS@L hydrogels, previously evaluated utilizing an early transcript factor of cardiomyocyte progenitor cells (Efraim et al., 2017; Rufaihah et al., 2017; Waters et al., 2018). Hydrogen ADSC-ASS@L was developed in cells which, after 5 days of incubation, greatly enhanced heart markers, namely Cx43, cTnl, and SAC. The results generally suggest the promising mitotic activity of the hydrogel ADSC-ASS@L as illustrated on Figure 4, for instance, the cardiac progenitor cells and cardiomyocytes. Furthermore, we agree that promising in vivo behavior with new injectable hydrogels for differentiation of the cardiomyocyte and proliferation should be exploited.

**Figure 5.**
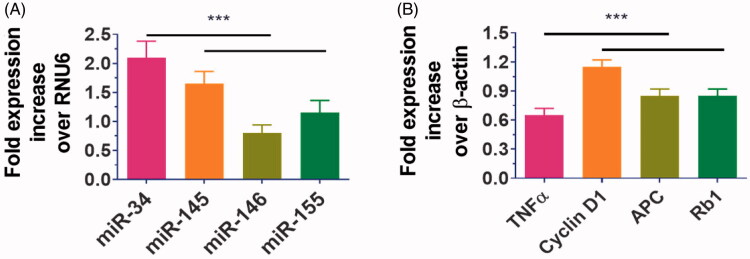
The gene expressions levels of the inflammatory markers genes and cardio stress investigated by qPCR analysis. ***p-value < .001.

Furthermore, saline and the community under treatment have been hydrodynamically evaluated. In the absence and in the presence of ADSCs after 28 days of treatment, no significant variations in body weights and cardiovascular ratios were identified between ADSC-ASS@L hydrogels. Increased cardiac outcomes, contracttility measures and LV strain were shown in the compare of hydrogels and saline treatment group ADSC-ASS@L and control units. The hydrogels were evaluated to be first in vivo cytocompatible and did not promote substantial immune responses to the animal model. As Figure 5(A,B) shows, research on myocardial tissue immunochemical bleaching have indicated that the cardiomyocyte and inflammatory factor (TNF-α) of ADSC-ASS@L hydrogels and of ASS@L hydrogel injections have not been obvious. Furthermore, when associated with untreated population, pro-inflammatory markers (TNFα and miR-146) and apoptotic genes, there was no significant alterations to the sample's cardiomyocyte (cyclin D1 and miR-145) and microRNA. We have therefore determined the synthesized cardiac renewal hydrogels from the ADSC-ASS@L.

**Figure 6.**
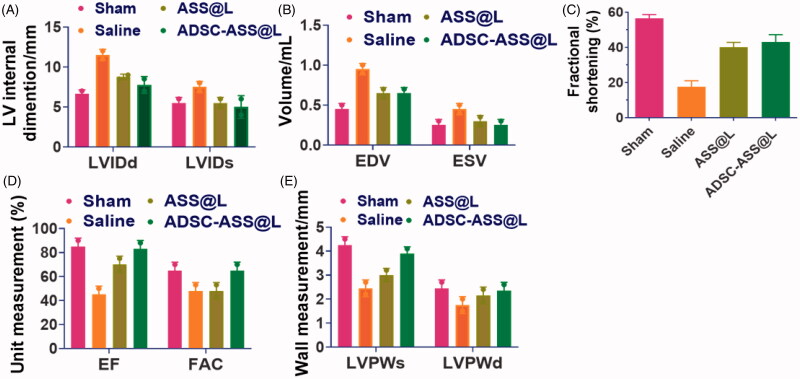
In vivo cardiac remodeling assessment after treatment with 28 days post administration of ASS@L and ADSC-ASS@L. (A) Left ventricular internal size of diastole and systole (LVIDd and LVIDs). (B) End diastolic and systolic volume (EVD and EVS) measurements. (C) Fractional shortenings of the ASS@L and ADSC-ASS@L. (D) Determinations of fractional area and ejection fraction change and Left ventricular posterior wall at diastolic and systolic sites.

In order to investigate the manner of ADSC activity following administration of heart illness after intramyocardial injections, ASS@L hydrogels were encapsulated with a myocardial infarction model for animals (Figure 6(A-E)). The LV mode reduced dramatically 30 days after past administrations, which is vital in cardiovascular disaster dissuasion. With myocardial attack groups the ADSC simplified hydrogels with ASS@L. In comparison to the hydrogelles and salines in the ADSC condensed hydrogels, the ejection fractions (EF: 70 ± 5.23) and the variance in the fractional areas (FA: 56.36 ± 4.1 were significantly elevated. In addition, the ASS @L hydrogels of the community generally greatly improve E-F and F-A change due to their active dynamic segments and their biocompatible qualities. Injectable diastole and systole (LVId) hydrogels were reinforced by the inter-ventricles ASS@L and saline hydrogels of 6.47 ± 0.60 and 5.05 ± 0.27 mm respectively. The treated ADSC-ASS@L community also shows an elevated end-substance (S) and end-diastolic (ED), which support cardiac operation of the injected hydrogels, with intensive amounts of 0.22++ 0.05 and 0.59 ± 0.04 mL. The left ventricula wall measures developed considerably in systoles and diastols after the ADSC-ASS@L treatment group in both pre- and post- ADSC comparison with control groups. The great results from our experiments will support the outstanding ADSC-encapsulated Hydrogels and the ASS@L hydrogels without ADSC, which are compatible with their biocompatibility and chemical dynamic assembly, with better cardiac functionality.

**Figure 7.**
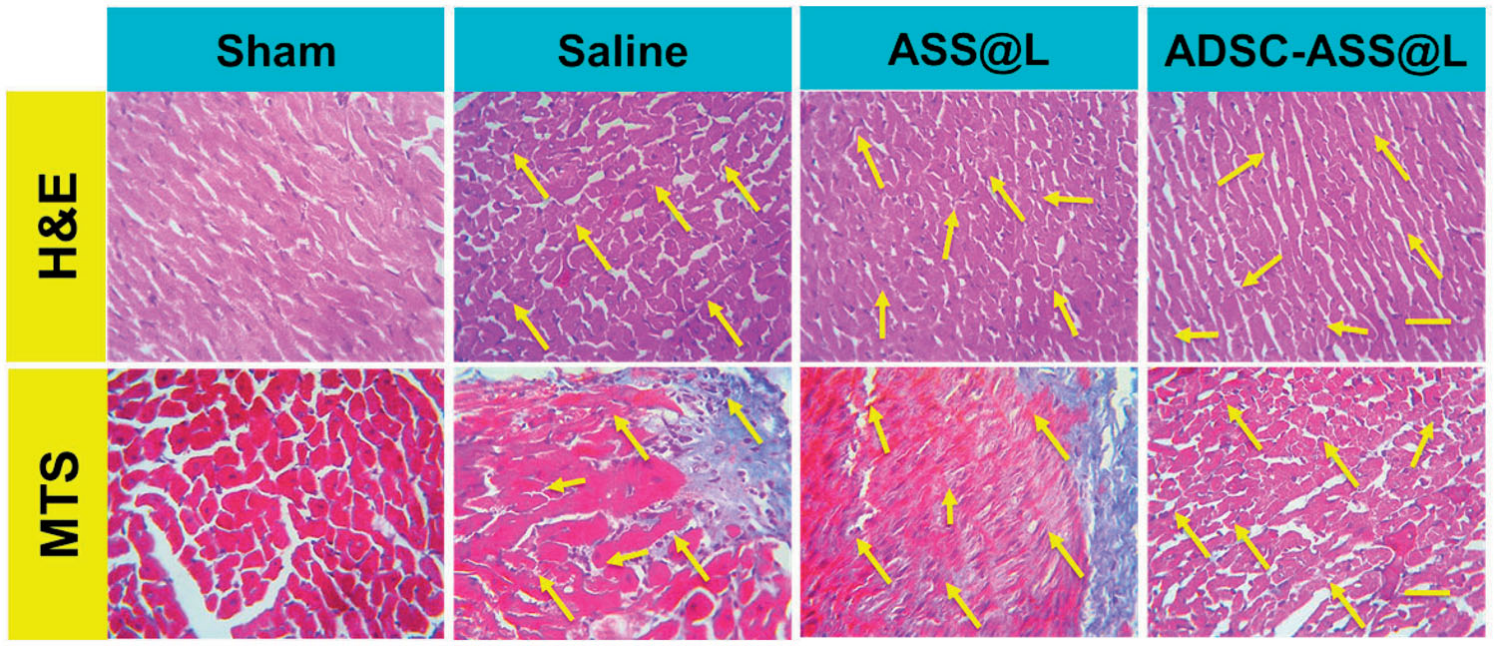
Regressions of myocardial fibrosis and cardiac hypertrophy in experiments. (A) Illustrative images of H&E staining and Masson staining (MTS) images of left ventricular tissue sections (magnification = 200×).

Cardiac hypertrophy and cardiac organ damage are the two characteristics of heart remodeling caused by excess pressure. In order to evaluate whether the melatonin has a therapeutic impact on these two situations a series of histological experiments were done using HE and Masson staining for the measurement of myocyte collage volume fraction (CCV) and transverse surface (CSA) in paraffin-embedded cardiac tissues. After eight weeks of big arteries after TAC surgery, levels of fibrosis and myocyte CSA increased considerably. CVF and CSA dropped substantially compared to the TAC population in all three treatments. Furthermore, the ADSC-ASS@L hydrogels group had slightly lower CVF and CSA than the other therapy groups (Figure 7).

**Figure 8.**
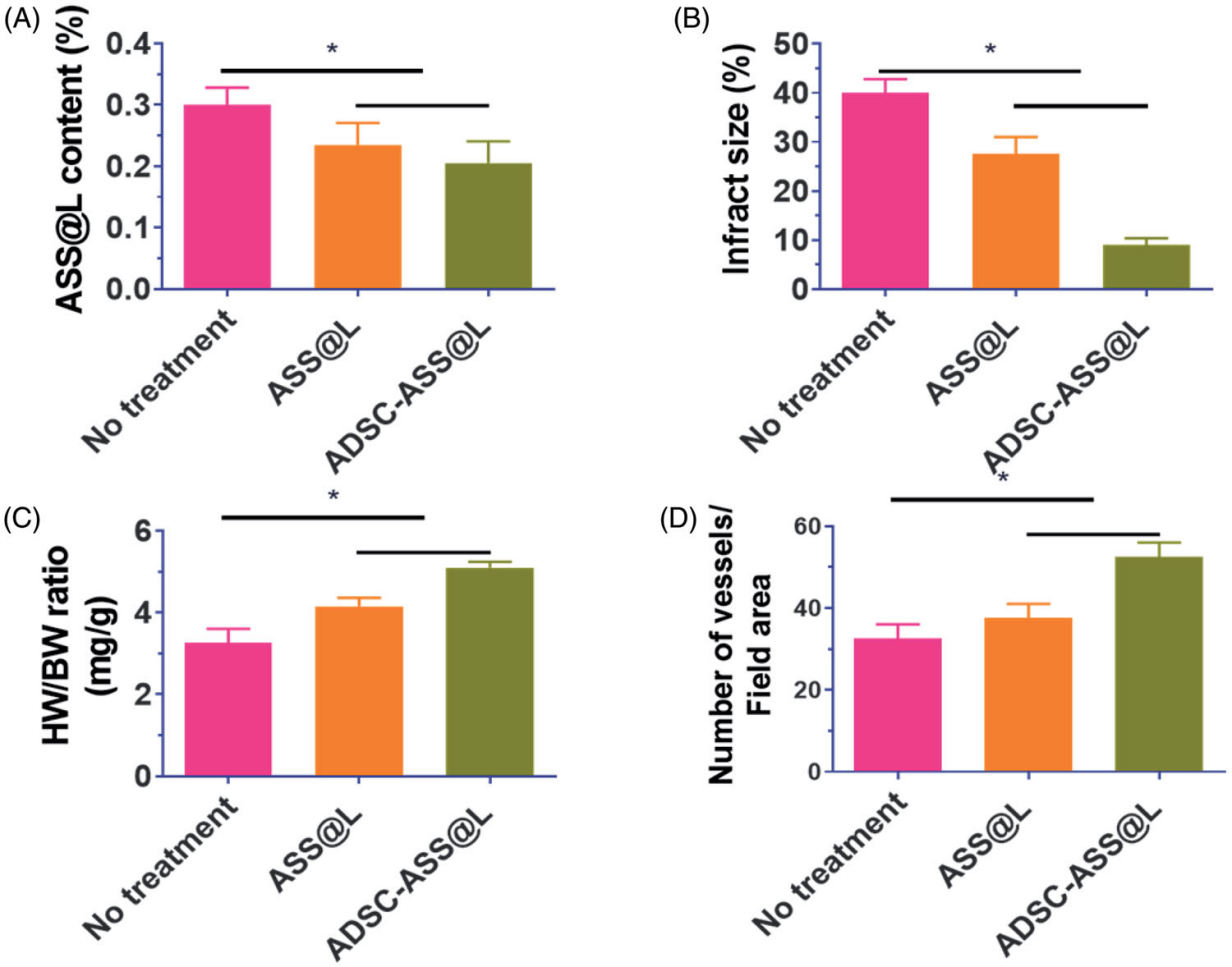
**(**A) ASS@L contents of infarct areas after hydrogels treatments with ASS@L and ADSC-ASS@L. (B) Measurements of infarct size of ASS@L and ADSC-ASS@L. (C) Examination of percentage of the heart weight (HW) to body weight (BW) after treatments of ASS@L and ADSC-ASS@L and (D) Number of the vesssels abd field area of ASS@L and ADSC-ASS@L. *p-value < .05.

After 28 days of post-administration, the region and dimensions of the patient party were semi contracted by the middle part of the papillary muscles (Thi et al., 2020; Sack et al., 2020; Domengé et al., 2021). In the ADSC-ASS@L hydrogels and control-treated classes, the successor wall width associated with the saline treatment community was later shown to establish cardiomyocyte survival of the per infarct portion. Thus, increased angiogenesis and reduced necrotic cardiomyocytes in hydrogels are crucial in enhancing cardiac use in models of myocardial infringements (Figure 8). Recently, scientists established ADSC therapy myocardial infringements, but there are various problems in transporting adipose stem cells into infarcted cardiac tissues. The study showed a significant impact on cardiac recovery and fractions of the sturdy hydrogels with separate adipose stem cells (Figure 8).

## 4. Conclusion

Simple ADSC-encapsulated hydrogetics were substantially created, similar to heart healing following AMIs. Physical and morphological evaluations reinforced the nanopolymeric substance's chemical interactions that promised the development of biomolecules. The precise hydrogels regulate anti-inflammatory factors in cardiomyocytic cells and cause anti-inflammatory action in vivo and in vitro experiments. Investigation was effectively conducted on the injecting of incapsulated ADSC hydrogules into the myocardial infarction site of the animal model, leading to projected healing benefits with enhanced vascular density and smaller myocardial infarction area expulsion fractions. Additionally, we ensured that these kind of hydrogels are important instrument for the potential treatment of cellular vesicles and myocardial infarctions in hypertrophy.
